# Diagnosis of Desmoplastic Reaction by Immunohistochemical Analysis, in Biopsy Specimens of Early Colorectal Carcinomas, Is Efficacious in Estimating the Depth of Invasion

**DOI:** 10.3390/ijms140713129

**Published:** 2013-06-25

**Authors:** Kazuya Ohno, Takahiro Fujimori, Yosuke Okamoto, Kazuhito Ichikawa, Takeshi Yamaguchi, Johji Imura, Shigeki Tomita, Hiroyuki Mitomi

**Affiliations:** 1 Department of Surgical and Molecular Pathology, Dokkyo Medical University, 880 Kitakobayashi, Mibu, Shimotsuga, Tochigi 321-0293, Japan; E-Mails: kaz-ohno@dokkyomed.ac.jp (K.O.); t-fuji@dokkyomed.ac.jp (T.F.); yokamoto@dokkyomed.ac.jp (Y.O.); i-kazu@dokkyomed.ac.jp (K.I.); take_eleven@yahoo.co.jp (T.Y.); sstomita@dokkyomed.ac.jp (S.T.); 2 Department of Gastroenterology, Shizuoka City Shizuoka Hospital, 10-93 Otemachi, Aoi-ku, Shizuoka City, Shizuoka 420-8630, Japan; 3 Division of Gastroenterology and Hepatology, Department of Internal Medicine, Toho University Omori Medical Center, 6-11-1 Omorinishi, Ota-ku, Tokyo 143-8541, Japan; 4 First Department of Surgery, Dokkyo Medical University, 880 Kitakobayashi, Mibu, Shimotsuga, Tochigi 321-0293, Japan; 5 Department of Diagnostic Pathology, Graduate School of Medicine and Pharmaceutical Science, University of Toyama, 2630 Sugitani, Toyama City, Toyama 930-0194, Japan; E-Mail: imura@med.u-toyama.ac.jp

**Keywords:** immunohistochemistry, biopsy, desmoplastic reaction, early colorectal carcinoma, submucosal invasion

## Abstract

The aim of our study was to evaluate the diagnosis of desmoplastic reaction (DR) by immunostaining for α-smooth muscle actin (αSMA) and desmin, for predicting the depth of submucosal invasion in biopsy specimens of early colorectal carcinomas (CRCs). Thirty-eight cases of non-pedunculated early CRCs were included in this study. Positive for DR was defined as αSMA-positive and desmin-negative stroma in the CRC. The depth of submucosal invasion was measured in endoscopically or surgically resected specimens and the lesions were subsequently divided into two groups: Group A (carcinoma *in situ*/intramucosal carcinoma and submucosal invasive carcinoma with a depth <1000 μm) and Group B (submucosal invasion with a depth ≥1000 μm). Twenty-one cases were DR-positive and 17 were DR-negative. No statistical significance was found between the DR with regard to tumor size, location and histological type. All DR-positive cases belonged to Group B whereas 14 (82.4%) DR-negative lesions belonged to Group A (*p* < 0.001). The sensitivity, specificity, positive and negative predictive values and accuracy of DR positivity for diagnosis of Group B were 87.5%, 100%, 100%, 82.4% and 92.1%, respectively. Conclusively, detection of DR in biopsy specimens with ancillary immunohistochemistry (αSMA/desmin) would help in preoperative diagnosis for the depth of submucosal invasion of early CRC.

## 1. Introduction

Early colorectal carcinomas (CRCs) were classified into three groups: M, carcinoma *in situ* and intramucosal carcinoma; SM1, submucosal invasive carcinoma with a depth of less than 1000 μm; and SM2, submucosal invasive carcinoma with a depth of greater than or equal to 1000 μm [1]. We previously demonstrated a relationship between the depth of submucosal invasion (SM depth) and the frequency of lymph node metastasis in resected early CRCs [2]. Endoscopic resection, including polypectomy, endoscopic resection and endoscopic submucosal dissection may be applied for M CRCs [3], pedunculated SM CRCs diagnosed as head invasion [4], and non-pedunculated SM1 CRCs [2,5]. Conversely, SM2 CRCs show lymph node metastasis in 10 to 15% of cases, and surgical resection accompanied by lymph node dissection is recommended [5]. The rate of lymph node metastasis is not thought to be related to SM depth in SM2 CRC [2]. Thus, discrimination between M/SM1 CRC (Group A) and SM2 CRC (Group B) is very important for selecting therapeutic modalities against SM CRC. We also previously reported that the detection of desmoplastic reaction (DR) in pretreatment biopsy specimens was useful for predicting the SM depth in non-pedunculated, but not in pedunculated SM CRCs [1,6]. In these studies, DR was evaluated by hematoxylin and eosin (H&E) staining of the biopsy specimens, although it was difficult to diagnose DR in some cases of pedunculated CRCs [1].

The aim of the present study was to evaluate the diagnosis of DR by immunostaining for α-smooth muscle actin (αSMA) and desmin for discriminating Group A from Group B in biopsy specimens of early CRCs.

## 2. Results and Discussion

### 2.1. Results

#### 2.1.1. Clinicopathological Features

Thirty-eight out of 51 (74.5%) early CRCs were of the non-pedunculated type and 13 (25.5%) were pedunculated. Pedunculated lesions were excluded in this study because in our preliminary analysis, DR by immunostaining for αSMA and desmin had no power to discriminate Group A from Group B [7]. Among the non-pedunculated type, 21 (55.3%) were DR positive lesions and 17 (44.7%) were DR-negative. No statistical significance was found between the DR positive and negative groups with regard to age, gender, tumor size, tumor location and histological type (Table 1).

#### 2.1.2. Relationship between Diagnosis of DR and the Depth of Invasion in Early CRCs

The immunohistochemical analysis for αSMA and desmin of biopsy specimens revealed that the DR-positive group was composed of 21 (100%) cases belonging to Group B, and the DR-negative group was composed of 14 (82.4%) Group A cases (*p* < 0.001) among the non-pedunculated type CRCs (Table 2). The calculated sensitivity, specificity, positive predictive value (PPV), negative predictive value (NPV) and accuracy of DR positivity to differentiate Group A from Group B were 87.5%, 100%, 100%, 82.4% and 92.1%, respectively.

### 2.2. Discussion

Desmoplastic reaction (DR), which is characterized by stromal fibrosis of invasive carcinomas, is thought to start increasing when carcinoma cells have invaded beyond the muscularis mucosae [8,9]. On the other hand, Martin *et al.* reported that the dense fibroblastic reaction is capable of restraining the progression of tumor cells and plays a role in tumor regression [10]. The differences in these articles may have arisen due to the complex crosstalk mechanisms between the carcinoma and stroma.

In the multicenter collaborative studies led by the *Japanese Society for Cancer of the Colon and Rectum* (JSCCR) [1,6], we previously demonstrated that the biopsy specimens from Group B were frequently evaluated as DR-positive lesions. These pathological examinations were performed on sections stained with H & E. Thus, these studies revealed that it is possible to predict SM depth by diagnosis of pretreatment biopsy specimens, especially in non-pedunculated, but not in pedunculated CRCs.

It is generally known that growth of myofibroblasts is affected not only by tumor progression but also by inflammatory or mechanical stimulation, and indeed, pedunculated colorectal tumors (such as pedunculated M CRC, Juvenile type polyp, Peutz-Jeghers type polyp and mucosal prolapse syndrome) tend to have abundant myofibroblasts around the muscularis mucosae, regardless of histological malignancy. In our former studies, we found that it was difficult to diagnose DR in some biopsy specimens from non-pedunculated CRCs that were stained with H&E. To resolve this problem, the present study was carried out to detect the presence of DR using biopsy specimens, immunostained for αSMA and desmin, and to determine whether or not the discrimination accuracy between Groups A and B could be improved.

In our retrospective study, the calculated sensitivity, specificity, PPV and NPV of DR positivity to differentiate Group A from Group B were 64.8%, 65.2%, 91.9% and 23.3%, respectively [6]. Subsequently, in our prospective study, the calculated sensitivity, specificity, PPV and NPV of DR positivity to differentiate Group A from Group B were 68.6%, 92.0%, 94.6% and 59.0%, respectively [1]. However, in this study, the calculated sensitivity, specificity, PPV and NPV of DR positivity to differentiate Group A from Group B were 87.5%, 100%, 100% and 82.4%, respectively. Thus, the sensitivity, specificity, PPV and NPV from the current study were markedly increased compared with the previously results. In particular, it was remarkable that DR-positive lesions were all Group B CRCs.

Although DR wasn’t detected in pretreatment biopsy specimens of the three cases in Group B, DR positivity was shown in the resected specimens from all of them. The endoscopic findings of them were all sessile. The mean tumor size of the three lesions was 20.7 mm (range, 20 to 22 mm), which was 7.7 mm greater than that of all 38 lesions. In retrospect, it seemed that inappropriate biopsies were performed in these 3 cases; inappropriate biopsies were defined as those taken at the edge of the tumor, but without the presence of SM2. Consequently, if the biopsies had included SM2 of the lesions, all Group B cases would have been accurately diagnosed with DR.

## 3. Experimental Section

### 3.1. Patients and Ethics

A total of 51 early CRCs in 49 patients, who had undergone surgical or endoscopic resection at Dokkyo University School of Medicine and its associated institutes between 1989 and 2004, were included in this study. They were classified endoscopically into pedunculated or non-pedunculated type as previously reported [2]. Non-pedunculated CRCs were selected as subjects of this study. Patients with inflammatory bowel disease and familial adenomatous polyposis were excluded from the study. The study was approved by the relevant institutional ethics committee in JSCCR and all samples were collected with the patients’ consent.

For the ethics procedure, a linkable anonymizing method was used to ensure the study was conducted in a blinded manner. Samples used in this study were materials from biopsy or surgery obtained for diagnosis or treatment and not for research purposes. Participation in the present study did not increase medical disadvantage or risk for patients, and data were used strictly for analysis of information as part of therapeutic intervention.

### 3.2. Histology and Immunohistochemistry

Specimens were immediately fixed in a 10% buffered formalin solution. Endoscopic resection specimens were cut at 2 mm intervals and surgically resected tissues were cut along the axis at 2 to 4 mm intervals for sections to be stained with H & E. The depth of the invasion, which was measured according to the previous report [2,11] was examined in H & E stained specimens and the lesions were subsequently divided into two groups: A and B. The histological type of the adenocarcinomas was subclassified according to the grade of differentiation, and the predominant pattern [11].

The biopsy specimens were stained with H & E and immunostained for αSMA and desmin. Immunohistochemical staining for αSMA and desmin was performed with an EnVision kit (Dako, Carpinteria, CA, USA) as described previously [12]. In brief, sections (4 μm thick) placed on silane-coated slides were deparaffinized, rehydrated, then placed in 0.01 M citrate buffer (pH 6.0) and treated by microwave heating (MI-77, Azumaya, Tokyo, Japan; 400 W, 95 °C) for 10 min to facilitate antigen retrieval. The slides were thereafter pretreated with 0.3% H_2_O_2_ in methanol for 20 min at room temperature to quench endogenous peroxidase activity. The sections were incubated with 1% bovine serum albumin in phosphate-buffered saline for 30 min, and then αSMA (clone 1A 4, Nichirei Biosciences, Tokyo, Japan) and anti-desmin (clone D33, DAKO, Denmark) mouse monoclonal antibodies for 60 min. Thereafter, the sections were incubated with polymer immunocomplexes for 60 min. Finally, the sections were incubated in 3,3′-diaminobenzidine tetrahydrochloride and then counterstained with Carrazzi’s hematoxylin.

### 3.3. Evaluation of Immunohistochemical Staining

**Figure 1:**
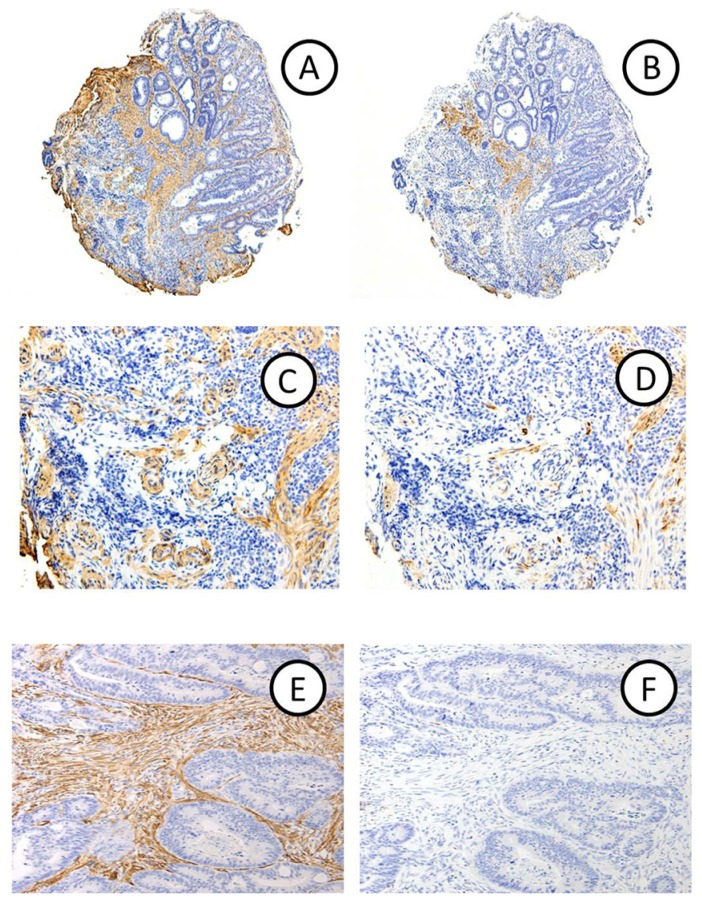
αSMA-positive (original magnification: A ×3; C ×100; E ×75) and desmin-negative (B ×3; D ×100; F ×75) DRs are seen in a biopsy (**A**–**D**) and a corresponding specimen obtained from surgically resected material (**E**,**F**) in a case of CRC with submucosal invasion (Group B).

Positive for DR in biopsy specimens was defined as αSMA-positive and desmin-negative stroma in the carcinoma. The vessel wall and nonspecific reaction were excluded from analysis. Representative micrographs of biopsy and resection specimens with DR are shown in Figure 1.

### 3.4. Statistical Analysis

Statistical analysis was done using the StatView J-5.0 program (SAS Institute Inc., Cary, NC, USA). Categorical analysis of variables was performed Chi-square test or Fisher’s exact test, as appropriate. For continuous variables, two-group comparisons were performed with the nonparametric two-sample Mann-Whitney U-test. All tests were two-tailed, with differences reported as significant if *p* < 0.05.

## 4. Conclusions

The detection of DR positivity in pretreatment biopsy specimens, immunostained for αSMA and desmin, is useful for discriminating Group A from Group B. The results suggest that the diagnostic accuracy for myofibroblasts, which are characterized by αSMA-positive expression and desmin negativity, is an important factor in evaluation of DR positivity in biopsy specimen stained with H & E.

## Figures and Tables

**Table 1:** Clinicopathological characteristics of 38 patients with non-pedunculated type of early colorectal carcinomas (CRCs).

	DR-positive	DR-negative	Total	*p*-value
Number of lesion, *n* (%)	21 (55.3)	17 (44.7)	38 (100)	

Age (years)				
Mean (range)	60.2 (39–79)	67.4 (53–79)	63.4 (39–79)	NS

Gender				
Male/Female, *n* (%)	12 (57)/9 (43)	12 (71)/5 (29)	24(63)/14 (37)	NS

Tumor Size (mm)				
Median (range)	20 (5–35)	13 (4–35)	16.5 (4–35)	NS

Location, *n* (%)				
Rectum	4 (19.0)	5 (29.4)	9 (23.7)	NS
Sigmoid	6 (28.6)	6 (35.3)	12 (31.6)	
Descending	2 (9.5)	1 (5.9)	3 (7.9)	
Transverse	4 (19.0)	5 (29.4)	9 (23.7)	
Ascending	4 (19.0)	0 (0)	4 (10.5)	
Cecum	1 (4.9)	0 (0)	1 (2.6)	

Histological type, *n* (%)				NS
Well-differentiated	14 (67)	15 (88)	29 (76)	
Moderately-differentiated	7 (33)	2 (12)	9 (24)	

DR, desmoplastic reaction; NS, not significant.

**Table 2:** Relationship between DR and the depth of invasion (Group A, B) of non-pedunculated early CRCs.

DR	Group A	Group B	*p*-value
Positive	0	21	<0.001
Negative	14	3	

DR, desmoplastic reaction.
